# Psychosocial Stressors Associated With Emotional and Behavioral Problems Among Street-Connected Adolescents in Dhaka, Bangladesh: A Cross-Sectional Study

**DOI:** 10.7759/cureus.113734

**Published:** 2026-07-31

**Authors:** Farjana Akter, Tanbir Ahmed, Taslima Begum, Mahabuba Rahman, Itfat K Disha, AHM Mostofa Kamal, Md. Selim Babu, Shelina Shahid, Nahid Morshed

**Affiliations:** 1 Child and Adolescent Psychiatry, Green Life Medical College and Hospital, Dhaka, BGD; 2 Paediatric Medicine, Bangladesh Shishu Hospital & Institute, Dhaka, BGD; 3 Child and Adolescent Psychiatry, Bangladesh Medical University, Dhaka, BGD; 4 Psychiatry, Medical College For Women and Hospital, Dhaka, BGD; 5 Psychiatry, Bangladesh Medical University, Dhaka, BGD; 6 Psychiatry, Jessore Upazila Health Complex, Jessore, BGD

**Keywords:** bangladesh, dhaka stress scale-adolescent, emotional and behavioral problems, psychosocial stress, street adolescents, strengths and difficulties questionnaire

## Abstract

Background

Adolescence is a vulnerable period for emotional and behavioural problems, and street-connected adolescents face cumulative psychosocial adversities that may heighten this risk. However, evidence on the relationship between psychosocial stress severity and emotional‒behavioural problems among street adolescents in Bangladesh remains limited. This study aimed to assess psychosocial stressors and emotional-behavioural problems among street adolescents in Dhaka, Bangladesh, and to examine their associations.

Methods

This cross-sectional study was conducted by the Department of Psychiatry, Bangladesh Medical University, among 106 street adolescents aged 11-17 years who were recruited through purposive sampling from Kamalapur and Tejgaon Railway Stations, Dhaka, between June 2024 and August 2025. Psychosocial stress was assessed via the Dhaka Stress Scale-Adolescent (DSS-Ad), and emotional‒behavioural problems were assessed via the Bangla Strengths and Difficulties Questionnaire (SDQ). Associations were examined using Pearson’s chi-square test or Fisher’s exact test, as appropriate, and factors independently associated with abnormal conduct problems were examined using multivariable binary logistic regression.

Results

The mean age was 13.79 ± 1.70 years, and 96 (90.57%) participants were male. Mild stress was the most common category, affecting 58 (54.72%) participants, followed by severe stress in 30 (28.30%) and moderate stress in 18 (16.98%). Conduct problems were the most prevalent abnormal SDQ domain, occurring in 32 (30.19%) participants. Stress severity was significantly associated with emotional and conduct problems (both p < 0.001). After adjustment, moderate stress (adjusted OR (AOR)=4.14, 95% CI: 2.15-17.79) and severe stress (AOR=7.16, 95% CI: 4.57-23.27) were independently associated with higher odds of abnormal conduct problems, indicating a graded association. Adolescents without a history of physical abuse had lower adjusted odds of abnormal conduct problems than those who had experienced physical abuse (AOR=0.25, 95% CI: 0.08-0.82).

Conclusions

Psychosocial stress severity was strongly associated with emotional and behavioural problems, particularly conduct problems, among street adolescents in Dhaka. These findings suggest that psychosocial stress and violence exposure may represent important areas for trauma-informed assessment and support in this hard-to-reach population. Larger, longitudinal, multicentre studies are warranted to confirm these associations.

## Introduction

Adolescence, which spans the ages of 10-19 years, represents a critical and developmentally vulnerable period during which emotional, cognitive, and social maturation converge [1]. Mental health problems emerging during adolescence include depression, anxiety, and behavioural disorders. These problems frequently persist into adulthood and may have substantial long-term consequences for educational attainment, social functioning, and quality of life. According to the World Health Organization (WHO), one in seven adolescents globally experiences a mental disorder, accounting for approximately 15% of the total global disease burden in this age group [1]. A recent analysis of the Global Burden of Disease Study 2021 estimated that the global prevalence of mental disorders among adolescents and young adults aged 10-24 years reached 278.98 million, with the age-standardised rate increasing by approximately 14% between 1990 and 2021 [2]. Depression, anxiety, and behavioural disorders, including conduct disorders, are among the leading causes of illness and disability in this age group [1], with conduct disorders alone accounting for approximately 20% of all adolescent mental disorders globally [3].

The United Nations Committee on the Rights of the Child has defined street-connected children as those whose world and livelihood depend upon what they can obtain from the street, encompassing those living, working, or spending a substantial part of their time on the street without adequate adult supervision or protection [4]. It is estimated that tens of millions of children globally live or work on the streets, with the majority residing in low- and middle-income countries (LMICs) [5]. Street adolescents are routinely exposed to multiple co-occurring stressors: extreme poverty, homelessness, family disruption, physical and sexual abuse, community and police violence, substance use, social exclusion, interrupted education, and economic exploitation [5,6]. These stressors rarely occur in isolation. Under the allostatic-load framework, repeated adaptation to chronic, cumulative stress produces physiological "wear and tear", so maladaptive outcomes become more likely with the number and duration of exposure [7].

Emotional and behavioural difficulties are commonly assessed across five dimensions: emotional problems, conduct problems, hyperactivity/inattention, peer relationship problems, and prosocial behaviour, which are routinely operationalised using validated screening instruments such as the Strengths and Difficulties Questionnaire (SDQ) [8]. Among street-connected youth, these difficulties are markedly elevated: a Nigerian study reported a current psychiatric-disorder prevalence of 57.7% among street-connected Almajiri children compared with 37.0% among school pupils, with higher levels of depression, substance use, and post-traumatic stress disorder [9]. Similarly, violence and maltreatment among Burundian street children have been associated with post-traumatic stress, disrupted schooling, and subsequent risks of educational failure, substance use, antisocial behaviour, and adult mental disorders [6].

Substantial evidence links psychosocial stress to emotional and behavioural problems through mechanisms such as hypothalamic-pituitary-adrenal axis dysregulation, trauma-related disruption of emotion regulation, and the cumulative effects of early adversity on developing stress-response systems, consistent with a developmental psychopathology perspective [7]. A systematic review from LMICs reported that the prevalence of emotional problems ranged from 1% to 41% and generally exceeded estimates from high-income settings, further highlighting psychosocial stress as a potentially modifiable and important intervention target [10].

Bangladesh illustrates these concerns. An estimated one to three million children live on the streets of Dhaka amid illness, violence, and poverty [11], and a 2024 national report estimated that more than 3.4 million children were in street situations countrywide without parental care [12]. However, child and adolescent mental health services remain scarce; most young people with mental health needs, especially in LMICs, cannot access care owing to low availability, cost, and stigma [13].

Research on street-connected youth in Bangladesh and South Asia has focused largely on substance use, violence, physical health, living conditions, or isolated psychiatric symptoms [11]. However, few studies have concurrently measured psychosocial stress severity and the full range of emotional and behavioural problems or examined their interrelationship in this group. Clarifying this link is essential for prevention, trauma-informed services, and evidence-based policy.

Street adolescents in Dhaka face cumulative psychosocial adversities that may contribute to emotional and behavioural difficulties; however, local evidence remains limited. Identifying stress-related determinants could inform targeted interventions, trauma-informed support, and evidence-based policy. Therefore, this study aimed to assess the severity of psychosocial stress among street adolescents in Dhaka. It also aimed to determine the distribution of emotional and behavioural problems in this population. Finally, the study examined the associations between psychosocial stress severity and emotional and behavioural outcomes.

## Materials and methods

Study design and setting

A cross-sectional study was conducted by the Department of Psychiatry, Bangladesh Medical University (formerly Bangabandhu Sheikh Mujib Medical University), Dhaka, Bangladesh. Field data collection was carried out at Kamalapur Railway Station and Tejgaon Railway Station in Dhaka between June 2024 and August 2025. These sites were purposively selected because they are major congregation points for street-connected adolescents in Dhaka, where many live or work as porters, vendors, waste collectors, or beggars. In addition, established non-governmental organization (NGO) outreach activities at these locations facilitated access to this hard-to-reach population and helped identify potentially eligible participants.

Study population

The study population comprised street adolescents aged 11-17 years of either sex who were living or working at the two study sites. This age range was selected to correspond with the validated Bangla version of the Strengths and Difficulties Questionnaire (SDQ) used in the study [8]. Adolescents who had been living or working on the street for less than six months or who had an acute medical or psychiatric condition at the time of approach were excluded.

Sampling procedure and sample size

A purposive sampling technique was used because street-connected adolescents constitute a highly mobile and hard-to-reach population for whom no complete or reliable sampling frame was available. This approach enabled the recruitment of accessible adolescents at the selected congregation sites who fulfilled the predefined eligibility criteria. The sample size was estimated using the standard formula for a single population proportion:

$n = \frac{Z^{2} \times p \times q}{d^{2}}$

where Z = 1.96 (95% confidence level), p = 0.27 (the proportion of street adolescents with emotional and behavioural problems reported in a previous Indonesian study) [14], q = 1 − p = 0.73, and d = 0.09 (margin of error). A 9% margin was adopted because a 5% margin produced an unfeasibly large requirement (n ≈ 310) for this hard-to-reach population. This yielded a minimum of 94 participants; allowing for 10% nonresponse, the target was 103. On the basis of availability during the study period, 106 adolescents were ultimately enrolled (70 from Kamalapur and 36 from Tejgaon).

Data collection

To access this hard-to-reach population, the principal investigator accompanied an NGO during its scheduled food-distribution visits, when adolescents commonly gathered at both study sites. The NGO’s role was limited to facilitating access, identifying potentially eligible adolescents, verifying their identity, and helping prevent duplicate enrolment. Eligibility and recruitment were determined according to the predefined study criteria, and participants were approached sequentially. Receipt of NGO services was not an eligibility requirement. Nevertheless, because recruitment was conducted during NGO outreach visits, the possibility of selection bias could not be completely excluded and was acknowledged as a study limitation.

Interviews were conducted by the principal investigator, who had received training in administering the Dhaka Stress Scale-Adolescent (DSS-Ad) [15] with permission and the Bangla version of the SDQ [8], communicating with vulnerable adolescents, recognising psychological distress, and following appropriate referral procedures when required. After the study aims, procedures, and instruments were explained, voluntary written informed assent was obtained in Bangla before each interview; thereafter, the researcher read a semi-structured sociodemographic questionnaire and both study instruments and recorded the responses. Each interview lasted approximately 30-35 minutes. The instruments were pretested on 30 adolescents outside the main sample. Each participant was assigned a unique identification number, and completed forms were stored securely, with access restricted to the principal investigator.

Assessment of psychosocial stress (DSS-Ad)

Psychosocial stress was assessed via the DSS-Ad, a culturally validated 56-item self-report instrument developed for Bangladeshi adolescents that assigns weighted life-change values to stressful events experienced over the preceding 12 months [15]. The scale demonstrated excellent content validity (item-level Content Validity Index (CVI) ≈ 1; scale-level CVI = 0.93), good concurrent validity (r = 0.72), and high internal consistency (α = 0.83-0.97). Total scores classify stress severity as mild (≤150), moderate (151-300), or severe (>300).

Assessment of emotional and behavioral problems (SDQ)

Emotional and behavioral problems were assessed via the self-reported version of the SDQ [8], a 25-item screening tool administered in its Bangla version, consistent with the age range (11-17 years) used for the DSS-Ad in the same study population [15]. Its five subscales, such as emotional problems, conduct problems, hyperactivity/inattention, peer relationship problems, and prosocial behaviour, were scored via established cutoffs, categorising each domain as normal, borderline, or abnormal, and the accompanying impact supplement assessed symptom-related distress and impairment over the previous six months [8].

Statistical analysis

The collected data were checked for completeness, coded, and entered into IBM SPSS Statistics for Windows, version 26.0 (Released 2018; IBM Corp., Armonk, New York, United States) for analysis. No missing values were identified among the variables included in the analysis; therefore, all 106 participants were included. Quantitative variables, such as age, were summarised as means with standard deviations, whereas categorical variables were presented as frequencies and percentages. Pearson’s chi-square test was used to examine associations between categorical variables, and Fisher’s exact test was applied when the assumptions of the chi-square test were not met. Variables showing significant bivariate associations with abnormal conduct problems were entered into a multivariable binary logistic regression model to identify independent factors associated with the outcome after adjustment for potential confounders. Multicollinearity among the independent variables was assessed using tolerance and variance inflation factor (VIF) values, with a tolerance value below 0.10 or a VIF above 10 considered indicative of problematic multicollinearity. The overall fit of the logistic regression model was assessed using the Hosmer-Lemeshow goodness-of-fit test. Adjusted odds ratios (AORs) with 95% confidence intervals (CIs) were calculated. All statistical tests were two-tailed, and a p-value of less than 0.05 was considered statistically significant.

Ethical considerations

Ethical approval was obtained from the Institutional Review Board of Bangladesh Medical University (approval number: BSMMU 2024/5670), and the study was conducted in accordance with the principles of the Declaration of Helsinki. Written informed assent was obtained directly from the participating adolescents themselves after the study procedures were explained to them in Bangla; participation was non-coercive and uncompensated. Due to the street-based nature of the population, many participants were not living with or accompanied by parents/guardians, and parental consent could not be obtained. Confidentiality and anonymity were maintained through coded data handling; the participants were free to withdraw at any stage without consequence, and the data were retained securely for five years.

## Results

A total of 106 street-aged adolescents were enrolled in this study. The mean age of the participants was 13.79 ± 1.70 years, with the majority falling in the 14-17 years age group (n=59, 55.66%). Male adolescents constituted an overwhelming proportion of the sample (n=96, 90.57%), whereas female adolescents accounted for only 9.43% (n=10). Nearly all participants identified as Muslim (n=104, 98.11%). Regarding living arrangements, most did not live with their families (n=89, 83.96%), although more than half of the participants maintained contact with their families (n=61, 57.55%). Most participants were employed as labourers (n=81, 76.42%), whereas nearly one quarter were involved in begging (n=25, 23.58%). Substance use was reported by 39 (36.79%) participants, and physical abuse was experienced by exactly half (n=53, 50.00%). In terms of education, 57 (53.77%) had no formal education, whereas 49 (46.23%) had completed some primary schooling. The majority reported income between 10,000 and 20,000 Bangladeshi Taka (BDT) (61.32%) (Table 1).

**Table 1:** Sociodemographic and psychosocial characteristics of the study participants (N=106) Data is given as frequency and percentage except for age, which is given as mean ± SD.

Variable	Category	Frequency	Percentage
Age (years)	Mean ± SD	13.79 ± 1.70	
Age group	11–13 years	47	44.34
	14–17 years	59	55.66
Gender	Male	96	90.57
	Female	10	9.43
Religion	Islam	104	98.11
	Hinduism	2	1.89
Contact with family	Yes	61	57.55
	No	45	42.45
Living arrangement	With family	17	16.04
	Others	89	83.96
Profession	Labor	81	76.42
	Beggar	25	23.58
Substance use	Yes	39	36.79
	No	67	63.21
Education	No formal education	57	53.77
	Primary	49	46.23
Physical abuse	Yes	53	50.00
	No	53	50.00
Income	<10,000 BDT	41	38.68
	10,000-20,000 BDT	65	61.32

With respect to psychosocial stress severity, mild stress was the most common category (n=58, 54.72%), followed by severe stress (n=30, 28.30%) and moderate stress (n=18, 16.98%). Among the SDQ domains, conduct problems had the highest prevalence of abnormal scores (n=32, 30.19%), followed by prosocial difficulties (n=22, 20.75%) and emotional problems (n=16, 15.09%). Peer problems were rated abnormal in 12 (11.32%) and hyperactivity in 10 (9.43%) participants (Table 2).

**Table 2:** Distribution of psychosocial stress severity and emotional-behavioral problems among the study participants (N=106)

Variable	Category	Frequency	Percentage
Stress severity	Mild	58	54.72
	Moderate	18	16.98
	Severe	30	28.30
Emotional problems	Normal	86	81.13
	Borderline	4	3.77
	Abnormal	16	15.09
Conduct problems	Normal	65	61.32
	Borderline	9	8.49
	Abnormal	32	30.19
Hyperactivity	Normal	93	87.74
	Borderline	3	2.83
	Abnormal	10	9.43
Peer problems	Normal	58	54.72
	Borderline	36	33.96
	Abnormal	12	11.32
Prosocial behavior	Normal	64	60.38
	Borderline	20	18.87
	Abnormal	22	20.75

Stress severity was significantly associated with emotional problems (χ²(4)=26.14, p<0.001, Cramér’s V=0.34) and conduct problems (χ²(4)=33.79, p<0.001, Cramér’s V=0.39). Among participants with severe stress, 12 (40.00%) had abnormal emotional problems, compared with two (3.45%) among those with mild stress. Similarly, abnormal conduct problems were observed in 19 (63.33%) participants with severe stress compared with four (6.90%) participants with mild stress. No statistically significant associations were observed for hyperactivity (p=0.072, Cramér’s V=0.20), peer problems (p=0.119, Cramér’s V=0.18), or prosocial behaviour (p=0.247, Cramér’s V=0.16) (Table 3).

**Table 3:** Associations between psychosocial stress severity and emotional-behavioral problems SDQ: Strengths and Difficulties Questionnaire

SDQ Domain	Stress Severity	Normal, n (%)	Borderline, n (%)	Abnormal, n (%)	χ² (df)	Effect Size (Cramér's V)	p-value
Emotional problems	Mild	54 (93.10)	2 (3.45)	2 (3.45)	χ²(4) = 26.14	0.34	<0.001
	Moderate	15 (83.33)	1 (5.56)	1 (5.56)			
	Severe	17 (56.67)	1 (3.33)	12 (40.00)			
Conduct problems	Mild	49 (84.48)	5 (8.62)	4 (6.90)	χ²(4) = 33.79	0.39	<0.001
	Moderate	8 (44.44)	1 (5.56)	9 (50.00)			
	Severe	8 (26.67)	3 (10.00)	19 (63.33)			
Hyperactivity	Mild	53 (91.38)	3 (5.17)	2 (3.45)	χ²(4) = 8.62	0.20	0.072
	Moderate	14 (77.78)	0 (0.00)	4 (22.22)			
	Severe	26 (86.67)	0 (0.00)	4 (13.33)			
Peer problems	Mild	26 (44.83)	25 (43.10)	7 (12.07)	χ²(4) = 7.35	0.18	0.119
	Moderate	14 (77.78)	2 (11.11)	2 (11.11)			
	Severe	18 (60.00)	9 (30.00)	3 (10.00)			
Prosocial behavior	Mild	30 (51.72)	12 (20.69)	16 (27.59)	χ²(4) = 5.40	0.16	0.247
	Moderate	14 (77.78)	2 (11.11)	2 (11.11)			
	Severe	20 (66.67)	6 (20.00)	4 (13.33)			

Table 4 presents the distribution of abnormal emotional and behavioural outcomes across selected variables. Compared with younger adolescents, older adolescents aged 14-17 years had significantly higher proportions of peer problems (n=11 (18.6%) vs. n=1 (2.1%), p=0.005, Cramér’s V=0.24) and prosocial difficulties (n=19 (32.2%) vs. n=3 (6.4%), p=0.002, Cramér’s V=0.26). Emotional problems were significantly more common among labourers than among adolescents involved in begging (n=16 (19.8%) vs. n=0 (0.0%), p=0.031, Cramér’s V=0.18). Exposure to physical abuse was significantly associated with abnormal conduct problems, which were observed in 24 (45.3%) adolescents with a history of physical abuse compared with eight (15.1%) adolescents without such a history (p=0.002, Cramér’s V=0.27). Substance use was significantly associated with emotional problems (n=10 (25.6%) vs. n=6 (9.0%), p<0.001, Cramér’s V=0.31), conduct problems (n=21 (53.8%) vs. n=11 (16.4%), p<0.001, Cramér’s V=0.33), and hyperactivity (n=7 (18.0%) vs. n=3 (4.5%), p=0.037, Cramér’s V=0.18). Lack of family contact was significantly associated with conduct problems (n=20 (44.4%) vs. n=12 (19.7%), p=0.009, Cramér’s V=0.22) and hyperactivity (n=8 (17.8%) vs. n=2 (3.3%), p=0.016, Cramér’s V=0.20).

**Table 4:** Comparison of selected sociodemographic, family and behavioral characteristics according to abnormal emotional‒behavioral problems Values are presented as n (%) unless otherwise stated. χ²: chi-square test; df: degrees of freedom; V: Cramér’s V

Variable	Category	Emotional problems	Conduct problems	Hyperactivity	Peer problems	Prosocial difficulties
Age group	11-13 years	9 (19.1)	13 (27.7)	5 (10.6)	1 (2.1)	3 (6.4)
	14-17 years	7 (11.9)	19 (32.2)	5 (8.5)	11 (18.6)	19 (32.2)
	χ² (df), p, V	0.35 (1), p=.553, V=.05	0.02 (1), p=.876, V=.01	1.17 (1), p=.280, V=.09	7.88 (1), p=.005, V=.24	9.63 (1), p=.002, V=.26
Profession	Beggar	0 (0.0)	6 (24.0)	2 (8.0)	1 (4.0)	5 (20.0)
	Labor	16 (19.8)	26 (32.1)	8 (9.9)	11 (13.6)	17 (21.0)
	χ² (df), p, V	4.68 (1), p=.031, V=.18	0.13 (1), p=.718, V=.03	0.29 (1), p=.587, V=.05	0.75 (1), p=.385, V=.08	0.02 (1), p=.895, V=.01
Physical abuse	Yes	9 (17.0)	24 (45.3)	5 (9.4)	6 (11.3)	11 (20.8)
	No	7 (13.2)	8 (15.1)	5 (9.4)	6 (11.3)	11 (20.8)
	χ² (df), p, V	0.39 (1), p=.535, V=.05	9.79 (1), p=.002, V=.27	1.55 (1), p=.213, V=.11	0.01 (1), p=.914, V=.01	0.02 (1), p=.877, V=.01
Substance use	Yes	10 (25.6)	21 (53.8)	7 (18.0)	7 (18.0)	9 (23.1)
	No	6 (9.0)	11 (16.4)	3 (4.5)	5 (7.5)	13 (19.4)
	χ² (df), p, V	12.41 (1), p<.001, V=.31	13.97 (1), p<.001, V=.33	4.35 (1), p=.037, V=.18	2.00 (1), p=.158, V=.12	0.52 (1), p=.473, V=.06
Contact with family	Yes	8 (13.1)	12 (19.7)	2 (3.3)	5 (8.2)	10 (16.4)
	No	8 (17.8)	20 (44.4)	8 (17.8)	7 (15.6)	12 (26.7)
	χ² (df), p, V	1.07 (1), p=.300, V=.09	6.88 (1), p=.009, V=.22	5.84 (1), p=.016, V=.20	0.68 (1), p=.410, V=.07	0.72 (1), p=.395, V=.07

After adjustment for potential confounders, psychosocial stress severity showed the strongest independent association with abnormal conduct problems (Table 5). Compared with mild stress, moderate stress was associated with more than four times higher odds of abnormal conduct problems (AOR=4.14, 95% CI: 2.15-17.79, p=0.003), whereas severe stress was associated with more than seven times higher odds (AOR=7.16, 95% CI: 4.57-23.27, p=0.001), indicating a graded association. Adolescents without a history of physical abuse had 75% lower adjusted odds of abnormal conduct problems than those who had experienced physical abuse (AOR=0.25, 95% CI: 0.08-0.82, p=0.022). Substance use (p=0.073) and lack of family contact (p=0.286) were not statistically significant in the adjusted model.

**Table 5:** Multivariable logistic regression analysis of factors associated with abnormal conduct problems Outcome: abnormal conduct problems, coded 'yes' = 1 and 'no' = 0. Reference categories: physical abuse = yes, substance use = yes, contact with family = yes, and stress severity = mild. AORs were adjusted for all variables shown in the model; p<0.05 was considered statistically significant. AOR, adjusted odds ratio; CI, confidence interval

Variable	β	AOR	95% CI	p-value
No physical abuse (ref: Yes)	-1.395	0.25	0.08-0.82	0.022
No substance use (ref: Yes)	-1.175	0.31	0.09-1.11	0.073
No contact with family (ref: Yes)	0.711	2.04	0.55-7.51	0.286
Moderate stress (ref: Mild)	1.317	4.14	2.15-17.79	0.003
Severe stress (ref: Mild)	1.969	7.16	4.57 – 23.27	0.001

## Discussion

This study examined psychosocial stress and its association with emotional and behavioural problems among street adolescents in Dhaka, Bangladesh. A substantial proportion of participants experienced moderate-to-severe psychosocial stress, and conduct problems were the most common abnormal SDQ domain, followed by prosocial difficulties and emotional problems. Increasing stress severity was significantly associated with emotional problems and conduct problems, whereas physical abuse, substance use, lack of family contact, and older age were associated with selected emotional‒behavioural outcomes in bivariate analyses. In the multivariable analysis, psychosocial stress severity showed the strongest independent association with abnormal conduct problems, with progressively higher odds observed across the moderate and severe stress categories.

The observed psychosocial stress burden reflects the profound adversity faced by street-connected adolescents. Street children in Dhaka commonly experience chronic deprivation, including inadequate food, shelter, healthcare, and education, alongside violence and social exclusion [16]. A study among male street adolescents in Dhaka similarly reported low-to-moderate resilience, with drug abuse and witnessing street violence as key associated factors, highlighting the cumulative impact of the street environment on psychosocial functioning [17]. These findings are consistent with cumulative stress frameworks, where multiple co-occurring adversities intensify the overall psychological burden. A recent Dhaka-based study among school-aged adolescents also revealed that psychosocial stressors are significantly associated with psychiatric morbidity, as measured by the SDQ, with severe stress being an important determinant of emotional and behavioural dysfunction [18]. Compared with school-going adolescents, who generally experience milder stress profiles, the greater stress burden in the present sample may reflect the chronicity and multiplicity of street-related adversities [19]. A systematic review from South Asia also identified poverty, low socioeconomic status, working status, and poor parental relationships as important determinants of adolescent mental health problems [20].

The high prevalence of abnormal conduct problems in this study exceeded rates reported in general adolescent surveys in Bangladesh, where conduct disorder prevalence was estimated at 21.72% [21]. This finding is also consistent with evidence that children from slum and disadvantaged urban settings have higher rates of serious behavioural problems than do children from more stable environments [22]. Conduct problems among street adolescents may partly represent maladaptive survival responses in contexts of poverty, violence, weak supervision, and social exclusion, a pattern also reported among street-connected youth in sub-Saharan Africa [5]. The co-occurrence of prosocial difficulties and emotional problems further indicates that mental health problems in this group are multidimensional, as also reflected in the South Asian adolescent mental health literature [20].

The significant associations between increasing psychosocial stress severity and both emotional problems and conduct problems were the central findings of this study. This finding is partially concordant with previous evidence from Bangladeshi studies showing that severe stress is associated with emotional disorders among adolescents [18]. Chronic stress may contribute to emotional and behavioural dysregulation through prolonged activation of stress‒response pathways, including hypothalamic‒pituitary‒adrenal axis dysregulation, which can impair emotional regulation and increase vulnerability to internalising and externalising problems [23]. In street adolescents, limited caregiving, a lack of protection, and an absence of mental health support may further intensify maladaptive coping and behavioural dysregulation. The absence of significant associations with hyperactivity, peer problems, and prosocial behaviour may suggest that these domains are influenced by broader neurodevelopmental, interpersonal, and contextual factors or may reflect limited statistical power.

Physical abuse was significantly associated with abnormal conduct problems in the bivariate analysis. In the adjusted model, adolescents without a history of physical abuse had 75% lower odds of abnormal conduct problems than those who had experienced physical abuse. This pattern indicated a lower burden of conduct problems among adolescents without a history of physical abuse; however, it should not be interpreted as evidence of a causal protective effect. This finding is consistent with longitudinal evidence showing that childhood physical abuse is associated with persistent externalising behavioural trajectories [24]. Substance use is associated with emotional problems, conduct problems, and hyperactivity, aligning with evidence linking adolescent substance use with conduct problems, hyperactivity/inattention, and reduced prosocial behaviour [25]. In Dhaka, substance use among street children has also been reported to impair cognition, emotion regulation, and behaviour [16]. A lack of family contact was associated with conduct problems and hyperactivity, which is consistent with the literature linking disrupted family relationships and parental separation with conduct and attention-deficit/hyperactivity disorder (ADHD) symptoms through reduced monitoring, emotional unavailability, and impaired behavioural support [26]. Older adolescents had greater peer and prosocial difficulties, possibly reflecting increasing peer salience during mid-to-late adolescence [27], compounded by cumulative stigma and social exclusion.

Multivariable logistic regression showed that psychosocial stress severity remained independently associated with abnormal conduct problems. Compared with mild stress, progressively higher adjusted odds were observed among adolescents with moderate and severe stress, indicating a graded association consistent with cumulative-risk models in developmental psychopathology [23]. Adolescents without a history of physical abuse had lower adjusted odds of abnormal conduct problems than those who had experienced abuse; however, this finding should not be interpreted as evidence of a causal protective effect. Substance use and lack of family contact were no longer statistically significant after adjustment, suggesting that their bivariate associations may overlap with other variables included in the model. Nevertheless, this finding does not negate previous evidence documenting a substantial burden of conduct problems among Bangladeshi adolescents [21] and associations between adolescent substance use and behavioural difficulties [25]. Overall, psychosocial stress and violence exposure warrant further investigation as potentially modifiable areas for trauma-informed support among street-connected adolescents.

A major strength of this study was the use of the DSS-Ad, a culturally validated and population-specific instrument developed for Bangladeshi adolescents, rather than a generic Western stress scale. The validated Bangla version of the SDQ also enabled culturally appropriate assessment of emotional and behavioural problems. However, several limitations should be considered. The cross-sectional, single-city design, modest sample size, purposive recruitment, and male-predominant sample limited causal interpretation and generalisability. Recruitment during NGO outreach visits may have introduced selection bias, while self-reported data were susceptible to recall and social desirability bias. Unmeasured factors and the limited number of outcome events may also have contributed to residual confounding and model overfitting; therefore, the regression findings should be interpreted cautiously. The findings have practical implications for services targeting street-connected adolescents. Existing NGO outreach and child-protection programmes may provide suitable entry points for trauma-informed screening, supportive counselling, violence-prevention initiatives, and referral for comprehensive mental health assessment. Particular attention should be given to adolescents experiencing severe psychosocial stress or physical abuse.

## Conclusions

Among street-connected adolescents in Dhaka, conduct problems were the most common abnormal emotional and behavioural outcome. Psychosocial stress severity was associated with emotional and conduct problems and with progressively higher adjusted odds of abnormal conduct problems. Adolescents without a history of physical abuse had lower adjusted odds of conduct problems than those who had experienced abuse. Because of the cross-sectional design, these findings do not establish temporality or causality. They suggest that psychosocial stress and violence exposure warrant attention in trauma-informed assessment and support for street-connected adolescents. Trauma-informed screening, counselling, violence-prevention measures, and referral pathways could be considered within existing outreach services. Larger, multicentre, longitudinal studies are needed to confirm these findings and evaluate relevant psychosocial interventions.
